# A Mixed-Methods Study Exploring Coping Self-Insights Associated with Resilience

**DOI:** 10.3390/bs14111018

**Published:** 2024-11-01

**Authors:** Kirsten J. Bucknell, Scott Hoare, Maria Kangas, Eyal Karin, Monique F. Crane

**Affiliations:** School of Psychological Sciences, Macquarie University, Sydney 2109, Australiamaria.kangas@mq.edu.au (M.K.); eyal.karin@mq.edu.au (E.K.); monique.crane@mq.edu.au (M.F.C.)

**Keywords:** resilience, coping self-insight, self-reflection, mixed methods, quantitization, inverse probability weighting

## Abstract

Self-insight has been associated with psychological resilience; however, less is understood about the role coping-specific self-insights play in strengthening resilience. This study used a convergent mixed-methods approach to investigate the coping self-insights triggered by self-reflection on coping experiences and their effects on perceived resilience. Australian ministry workers (*n* = 79) provided up to five qualitative self-reflective workbook entries, and quantitative online self-report survey responses before and six months after training. Hierarchical regression analyses of weighted quantized coping-specific self-insights on perceived resilience were conducted. Results suggest two pathways for the strengthening of resilience. A set of three self-insights related to greater perceived resilience appear to reinforce and sustain resilient beliefs across six months to increase perceived resilience. Another set of four self-insights is related to lesser perceived resilience after six months. It is suggested that the first set of self-insights may enhance beliefs that support resilience in the mid-term, whereas the second set may promote self-awareness that reduces perceived resilience in the mid-term. These findings support further exploration of coping self-insights, and the use and on-going testing of self-reflection resilience training.

## 1. Introduction

The role of specific self-insights (akin to a psychodynamic definition of an insight which is a conscious shift in meaning involving new associations) in facilitating mental health is somewhat ambiguous [1]. Researchers have explored how generalized self-insight (characterized by having a clear grasp of one’s thoughts, emotions, and behaviors [2]) contributes to the link between self-reflection and resilience; however, little is known about the effects of coping-specific self-insights [3,4]. Self-reflection is conscious introspection to understand one’s emotions, thoughts, and actions [2], whereas resilience is the maintenance of psychological function amidst a stressor experience [5]. Theoretical work presented as the Systematic Self-Reflection model of resilience strengthening proposes that when self-reflection on one’s coping process results in self-insights, opportunity exists to refine and enhance capacities for resilience in support of resilient outcomes [6]. However, the association between self-reflection and positive mental health outcomes such as resilience is mixed. Self-reflection can be adaptive by making sense of our experiences, enhancing adaptive emotion regulation, and providing new perspectives into difficult situations [7,8]. Conversely, self-reflection can take the form of maladaptive ruminative or perseverative thinking that can undermine mental health [9]. Initial studies suggest that for resilient outcomes to be attained from self-reflection, self-insight must occur [2,3]. However, scholars argue that not all self-insights are important to the development of capacities for resilience, and that outcomes vary based on factors such as the content and timing of the specific self-insight attained [1,10].

At a granular level, specific self-insights may encompass various metacognitions, ranging from understanding why a particular emotion has occurred, to more complex processes such as assigning meaning to a behavioral pattern [11]. Recent work suggests that specific coping self-insights are derived from reflection on everyday coping (e.g., recognizing effective strategies for specific situations), and are more predictive of resilience than generalized self-understanding [12]. Attainment of specific coping self-insights are thought to increase the likelihood of future resilient outcomes by promoting adaptation to capacities for managing daily stressors that need attention and action in a particular context (e.g., ineffective coping, triggering the identification of new strategies) [13]. However, there is currently no research establishing a link between these coping self-insights and psychological resilience.

### 1.1. Strengthening Resilience via Focused Self-Reflection and Coping Self-Insight

A growing body of research demonstrates that self-reflection on moderately stressful experiences has the potential to increase the likelihood of resilient outcomes [14]. A recent trial, from which this study is derived found that structured self-reflection on coping with stressor events increased perceived resilience compared to writing about the stressor events [15]. Theoretically underpinning that trial, the Systematic Self-Reflection model [6] identifies five practices that promote self-insight: (1) becoming self-aware of responses to stressors, (2) identifying stress triggers, (3) considering learning and development goals, (4) evaluating coping effectiveness, and (5) determining improvements to future coping efforts. These five self-reflective practices are proposed to engender specific coping self-insights that enable the development and refinement of an individual’s repertoire of resilient capacities, including resilient beliefs (e.g., coping self-efficacy, hope), the flexible application of coping strategies, and coping enabling resources (e.g., social networks).

### 1.2. Identification of Coping-Specific Self-Insights

Building upon the Systematic Self-Reflection model [6], the Self-Reflection and Coping Insight Framework [13] was introduced to articulate coping-related reflections and self-insights. Previous qualitative work has provided evidence for 14 insights emerging from the self-reflection process, but there was an acknowledgement of a need to refine the framework via empirical work [12]. The current study employs the framework to identify, quantify, and examine the relationship between these specific coping self-insights and perceived resilience.

### 1.3. The Present Study

Secondary to the aims of the parent trial [15], the purpose of this study was to understand if the attainment of specific coping self-insights through self-reflection was differentially associated with perceived resilience. That is, would singular coping self-insights be positively, negatively, or not significantly associated with perceived resilience. The present study uses a mixed-methods approach adopting a critical realist metatheoretical position, thus removing the potential philosophical incompatibility of integrating both qualitative and quantitative methods of data collection [16]. By merging a qualitative dataset that captures the introspective process that leads to gaining self-insights and describes the nature of specific coping self-insights [1,12] with a quantitative dataset of participant outcomes, we sought to understand the relationship between specific coping self-insights and participant outcomes. To test this, we codified qualitative coping self-insights achieved through self-reflection and examined their relationship to quantified perceived resilience. As research in this area is nascent, we tentatively hypothesized that perceived resilience would be associated with attainment of coping self-insights through self-reflection.

## 2. Materials and Methods

### 2.1. Openness and Transparency

This study was part of a randomized controlled trial of a self-reflection-based resilience strengthening training program [15]. It was not pre-registered. This article analyzes previously unreported data that were collected in participant workbooks. The overlap between measures used in this analysis is presented in Table S1 for transparency.

### 2.2. Design and Participants

**Figure 1:**
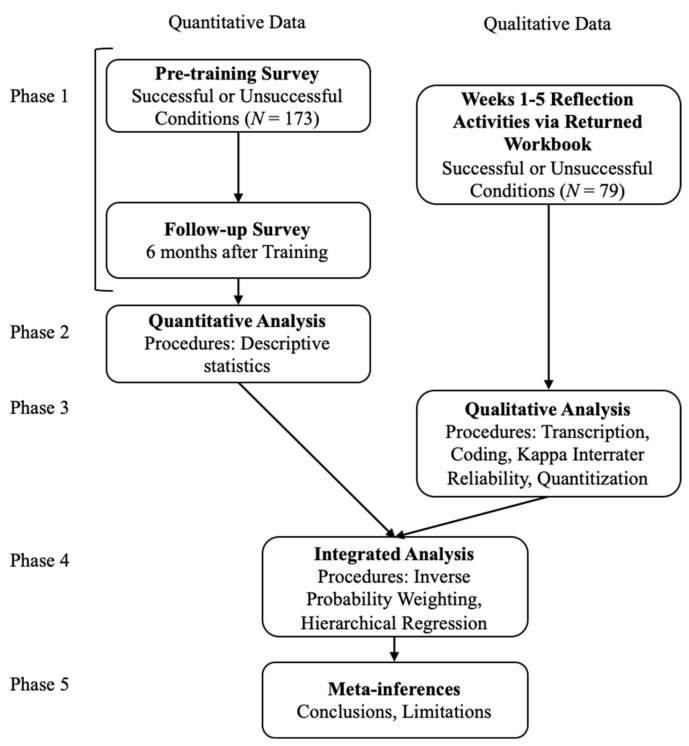
Convergent Mixed-Methods Intervention Design.

This study uses a convergent mixed-methods design (Figure 1). In the trial, a workbook (one for each self-reflection condition) was provided to participants to record a structured self-reflection once a week for five weeks [15]. See Supplementary Materials for example workbook questions and responses. Qualitative data were thus collected during the intervention to understand the introspective process of self-reflection resulting in attainment of coping self-insights. These data from self-reflection workbooks were transformed into numeric codes (i.e., quantitized) to assess coping self-insights. The quantitized data were then analyzed with quantitative data collected before and 6 months after the resilience training intervention.

The single inclusion criterion was that participants confirmed that they were Australian-based Protestant Christian ministry workers. They were recruited through social media posts, email distribution networks, and personal contacts of the lead author from March to July 2020. Participants registered for one of four research cohorts, with two cohorts starting in May 2020, one in July 2020, and the final cohort commencing in August 2020. Of the 254 ministry workers who consented to participate in the resilience training, 173 (68.11%) were randomly assigned to one of two self-reflection-based conditions in the trial (i.e., self-reflection on either successful or unsuccessful coping events). A third non-reflection control condition was not included in this analysis. All participants were invited to return their workbooks for analysis at the end of training. A total of 79 participants from the self-reflection conditions (46%) did so and were thus eligible for inclusion in the present study. There may be a range of reasons for this low return rate of workbooks, including effort required, a desire to retain workbook information or reflections for future use, or a desire to keep highly personal reflections private. This might have introduced bias into the sample as non-returning participants may have attained more, or different, self-insights. Participants in the sample for analysis (*n* = 79) were 63% male and 37% female, with a mean age = 46.23 years, (*SD* = 11.69, age range = 21–71 years) and had served in a formal ministry capacity for an average of 12.22 years (*SD* = 9.82, range 1–40 years).

### 2.3. Phase One: Intervention Procedure

Within the intervention trial, participants were randomly assigned to two self-reflection training conditions or a non-reflection control group (not used in the current study). Prior to commencement of this study, 510 participant identification numbers were assigned a number (1 = successful condition, 2 = unsuccessful condition, or 3 = control group) using an online random number generator (https://www.randomizer.org/). Participants were assigned an identification number, and therefore condition, based on their registration order. Participants watched a 35 minute online briefing, provided written consent, and completed pre-training tasks, then answered the same 10 workbook questions about a relevant stressor once each week for five weeks (see [15] for details). Questions were based on the five self-reflective practices outlined in the Systematic Self-Reflection model [6]. The two self-reflection conditions differed only in that the focus of participants’ reflection was directed either to a stressor that was managed *successfully* (S) or *unsuccessfully* (U). Adherence to instructions was evident in each condition (See [15]). Qualitative data was collected by hardcopy workbooks containing information on resilience and reflective tasks. Of the 79 participants in the self-reflection conditions who returned their workbooks, 77 (97%) completed their reflective questions in week 1, 74 (94%) in week 2, 68 (86%) in week 3, 61 (77%) in week 4, and 53 (67%) in week 5. The average number of weeks completed was 4.22.

### 2.4. Phase Two: Quantitative Measures

Quantitative data for this analysis were collected via an online survey at pre-training and 6 months post-training.

#### 2.4.1. Perceived Resilience

The Brief Resilience Scale (BRS) [17] measured perceived capacity to bounce back from hardship. Consisting of six items (e.g., “I tend to bounce back quickly after hard times”), participants were asked to indicate the extent to which the statements were reflective of their experiences in the previous three months on a 5-point scale from 1 (strongly disagree) to 5 (strongly agree). Analysis of this sample demonstrated strong internal reliability at both pre-training and 6-months post-training time points (⍺_Pre_ = 0.88), (⍺_+6months_ = 0.88).

#### 2.4.2. Self-Insight

Generalized self-insight was assessed pre-training using the insight subscale of the Self-Reflection and Insight Scale [2]. This factor measures clarity of self-understanding (e.g., “I usually have a very clear idea about why I’ve behaved in a certain way”) with eight items. As previous work has demonstrated low loadings, one item (i.e., “I am usually aware of my thoughts”) was removed from analysis [3,18]. Participants indicated their agreement with each statement on a 6-point scale from: 1 (strongly disagree) to 6 (strongly agree). Internal reliability was satisfactory in this sample (⍺_Pre_ = 0.78).

### 2.5. Phase Three: Qualitative Analysis

#### 2.5.1. Professional Contexts of Coders of Qualitative Data

The lead coder and first author [K.J.B.], an experienced organizational psychologist in Australian churches, provided valuable community-specific knowledge. However, it was necessary for her to examine tacit biases and assumptions and to have these challenged by the other authors during coding. The second coder [S.H.], a post-graduand in organizational psychology, had expertise in applying the coping insight Framework to qualitative data. The final coder [M.F.C.], a psychologist, contributed a decade of experience in resilience-focused research on coping insight and reflection processes.

#### 2.5.2. Analysis of Qualitative Data

Coping self-insights were assessed qualitatively and then transformed via quantitizing into quantitative data [19]. Qualitative data taken from participant workbooks were assessed using a deductive/inductive hybrid thematic analysis increasingly seen in mixed-methods research using convergent designs [20].

Starting with a deductive approach, coping self-insights were identified within participant workbook entries and coded using the 14 insights detailed in the Self-Reflection and Coping Insight Framework [12,13]. Handwritten workbook entries were transcribed. The first author read all workbook entries (333 weekly entries from 79 participants) and coded all identified self-insights according to those listed in the Self-Reflection and Coping Insight Framework [12,13]. An insight was differentiated from a lesson as self-knowledge or contextual understanding that was global in nature, i.e., it had a broader perspective than descriptive reports of behavior, thoughts, or feelings during a particular coping event [21]. Insight was also differentiated from self-reflection. Self-reflection is the conscious activity of thinking about past events (e.g., stressors) as opposed to the self-knowledge gained from reflective practice [13]. Narratives that suggested attainment of a self-insight but that did not reasonably fit a category were coded ‘other’.

The second author independently coded a random selection of half the workbooks (165 weekly entries (49.5%) from 40 participants (50.6%)) using the same method as the first author. Weekly meetings were held between these two authors to review code definitions and discuss interpretation of specific cases. The final author was consulted bi-weekly to review a sample of codes (∼10%) and resolve coding differences. The involvement of multiple coders was intended to reduce bias associated with any one coder’s perspective.

Intercoder agreement was assessed for each of the 14 coping self-insights with a target kappa score of 0.6 or greater (indicating good agreement or more) [22]. Several iterations of coding followed by disagreement resolution between the coders were undertaken to attain the kappa target for all 14 self-insights. Coding was concluded with the final author acting as arbiter to agree on definitional adjustments and resolve any outstanding disagreements. Final kappa scores ranged from 0.639 to 0.851.

Then overlaying an inductive approach, review was made of self-insights that did not meet the definition of one of the framework’s self-insights and had been classified as ‘other’. Initial codes were generated for ‘other’ self-insights as they were first identified. Within the inductive process, thematic analysis was applied by the first author to generate clusters of codes and to propose new themes and definitions [23]. All coders engaged in collaborative discussions to determine if there was substantial evidence to support a new theme. This involved assessing whether the new theme met the definition of an insight (as opposed to a reflective activity or lesson), determining that it was distinct from existing insights, and then establishing its definition in relation to existing scholarship.

Finally, to quantitize the data, each of the coping self-insights in the framework were coded as present (=1) or absent (=0) for each of the five weeks of participants’ self-reflective activities. A coping self-insight instance was: the demonstration of a defined coping self-insight in any of the five weeks of activity (where 0 = did not demonstrate that coping self-insight; 1 = did demonstrate that coping self-insight across the five weeks).

### 2.6. Phase 4: Integrated Analysis of Qualitative and Quantitative Data

The relationships between coping self-insights and perceived resilience were examined with a series of steps.

In the first step, descriptive statistics and correlations were reviewed for quantitative and quantitized data. Binary logistic regressions were conducted for condition (predictor variable) on each coping self-insight (outcome variable) to test if condition affected attainment of each coping self-insight.

In the second step, the statistical association between the coping self-insights attained throughout the intervention and perceived resilience were tested. Separate hierarchical linear regression analyses were conducted in the Statistical Package for Social Sciences (SPSS v28) for each of the coping self-insights to investigate if they predicted perceived resilience six months after training.

In the third step, consideration was given to the idea that although an attained coping self-insight might be strongly associated with resilience, its observed rarity might mask its statistical significance. That is, although a self-insight was rarely attained, it may yet be highly associated with perceived resilience. Therefore, to control for differences in occurrence and to increase statistical power, inverse probability weighting was applied, a common propensity score weighting adjustment method to assess the effects of exposure [24,25]. This technique is considered to oversample some cases artificially (i.e., rarely attained self-insights) but simulates the hypothetical situation where different coping self-insights are equally observed across the sample (i.e., balancing the sample for each model separately). The addition of a survey weight for each covariate is considered to add a “double robustness” for the exploration of observed relationships of interest [25]. Thus, the hierarchical linear regressions of the first step were re-examined with survey weights designed to oversample under-represented cases.

### 2.7. Phase 5: Meta-Inferences Drawn from Integrated Data

Adopting a mixed-methods approach, this study sought to integrate qualitative data extracted from workbooks (i.e., written narratives) with quantitative (i.e., perceived resilience) data to understand the associations of specific self-insights with perceived resilience. This was achieved by the conversion of qualitative data by quantitization (i.e., coping self-insight instances). Although quantitization is a considered a sensible mixed-methods approach that can demonstrate complexity through the merging of data sets, conversion of qualitative into quantitative data can also lead to the loss of the richness of narrative data [19]. Hence, inferences in this study were drawn by conducting not just deductive but also inductive thematic analysis, thereby identifying new themes that recognize the complexity of the data, as well as by using simplified, quantitized self-insight data as predictor variables in statistical analysis with quantitative perceived resilience data as the outcome variable [19]. Interpretation of integrated analyses was also made with reference back to qualitative workbook data.

## 3. Results

### 3.1. Phase 1: Intervention

The intervention was conducted as described.

### 3.2. Phase 2: Quantitative Descriptive Statistics

Quantitative data were collected prior to training and six months after the conclusion of training. Descriptive analyses and correlations are included in Supplementary Materials (Tables S2 and S3). No noteworthy patterns emerged.

### 3.3. Phase 3: Qualitative Analysis of Coping Self-Insights

In this phase, participant workbooks were analyzed to examine the coping self-insights evident in entries, as described by the Self-Reflection and Coping Insights Framework [12,13]. A summary, including definitions, examples, instances and rank order, is provided in Table 1. In line with a hybrid thematic analysis approach, definitional adjustments (to *Capacities applied* and *Capacity effectiveness*) and additions (i.e., *Capacity modification*) were made during coding (as described below).

#### 3.3.1. Adjustments Made to the Framework

The *Capacities applied* self-insight was originally defined as “acknowledging the diverse range of coping strategies, resources, or beliefs applied during the coping process” [13] (p. 8). Ministry workers never focused on the range of coping strategies applied but did acknowledge coping strategies that they typically use when coping with stressor events across time and contexts, for example “I can *tend* [emphasis added] to remove myself from relationship when stressed […] and become narrowly focused on task over people” (P205, S). Hence, the adjusted definition applied for this coping self-insight removed the requirement for specific identification of a diverse range of resilient capacities and became a recognition of global patterns of coping strategy use, beliefs engaged during stressor experiences, or application of resource usage, i.e., *acknowledging the coping strategies*, *resources*, *or beliefs typically applied in the coping process*.

*Capacity effectiveness*, originally referring to understanding “the nuanced interactions between stressor characteristics and the effectiveness of coping strategies or resources” [13] (p. 10), was adjusted to be defined more broadly in this study. Falon and colleagues [13] found that participants in their study most often considered contextual factors (e.g., lack of time, high control environment) that hampered the effectiveness of a strategy or resource. In the current study, participants regularly identified how effective resilient capacities were but did not associate them with the characteristics of the faced stressor or the interaction. The definition was therefore broadened to be more inclusive and to capture any insights relating to capacity effectiveness: *understanding the effectiveness of particular coping strategies*, *resources or beliefs for personal coping*. That is, having an insight about which resilient capacities work for the individual (or do not work) when dealing with a stressful situation (e.g., “Calling for advice from trusted people outside of the decision is very helpful” (P251, S)).

#### 3.3.2. Additional Insights Identified for Inclusion in the Coping Self-Insights Framework

After thematic analysis of ‘other’ self-insights, a new coping self-insight was identified as falling within the fifth self-reflective process ‘Future-Focus’. It is identified within Table 1 as *Capacity modification: understanding aspects of resilient capacities (*e.g., *skills*, *resources) that can be modified to support resilience in the future*. This self-insight captured identification of gaps in a participant’s current coping repertoire that required development of a new skill to become resilient to similar situations in the future (e.g., ‘I recognized a need for conflict resolution skills’ (P022, U)) or development in the effective execution of an attempted coping strategy (e.g., ‘After previous weeks I read about breathing exercises—they helped a bit […] but I need to be better at this’ (P136, S)). Others recognized a gap in the diversity of their coping repertoire but did not provide commentary on how they would meet their perceived need (e.g., “I’ve learned that I need a way to reduce my stress-levels in the moment of stress, not just afterward” (P159, U)).

#### 3.3.3. Participant Attainment of Coping Self-Insights

After the above adjustments and additions were made to the coding framework, analysis of coping self-insights contained within the workbook entries revealed that self-insights with the highest and lowest number of instances (or participants attaining that self-insight across the five weeks of self-reflective activity) were spread across the five reflective practices: *Capacity effectiveness* (*n* = 77; Evaluation), *Personal values* (*n* = 75; Self-awareness), and *Anticipated effect* (*n* = 73; Future-focus); and *Strengths* (*n* = 2; Evaluation), *Growth reappraisal* (*n* = 12; Reappraisal), and *Time course* (*n* = 12; Self-awareness).

## 3.4. Phase 4: Integrated Analyses

The purpose of this study was to explore if specific coping self-insights are differentially related to perceived resilience. It was hypothesized that perceived resilience would be associated with attainment of coping self-insights through self-reflection. Following review of descriptive statistics and correlations, binary logistic regressions were conducted to determine the effects of condition on each coping self-insight (Table S4). Generally, there were no condition differences for self-insight type. However, inflated standard errors were found for outcome variables *Capacity effectiveness* and *Strengths*, indicating an imbalance in the distribution of these two self-insights in the non-weighted data. For these two analyses, the effects cannot be accurately determined.

The hierarchical regressions with inverse probability weighting applied (see Table S5 for weighted sample sizes and Table S6 for coefficients of all regressions conducted) were used to explore whether perceived resilience 6 months post-reflection was related to the attainment of self-insight types when controlling for age, gender, self-reflection condition, pre-training general self-insight, and pre-training perceived resilience. Partially supporting the hypothesis, coping self-insights *Time course*, *Growth reappraisal*, and *Strengths* were significantly positively related to resilience 6 months post-training (t = 2.40, *p* = 0.017, β = 0.10; t = 3.58, *p* < 0.001, β = 0.18; t = 10.89, *p* < 0.001, β = 0.88, respectively), whereas contrary to the hypothesis, coping self-insights *Interpersonal effect*, *Capacities applied*, *Trigger interpretation*, and *Distinct outcomes* were negatively related to resilience 6 months post-training (t = −2.14, *p* = 0.034, β = −0.10; t = −2.09, *p* = 0.038, β = −0.10; t = −2.60, *p* = 0.010, β = −0.13; t = −3.62, *p* < 0.001, β = −0.17, respectively). Further, results for *Capacity modification* approached significance (t = −1.91, *p* = 0.057, β = −0.10). Moreover, the remaining seven coping self-insights (i.e., *Stressor reactions*, *Personal values*, *Trigger patterns*, *Capacity effectiveness*, *Desired responses*, *Anticipated effect*, and *Resource congruence*) were not significantly associated with perceived resilience.

### 3.4.1. Coping Self-Insights Positively Related to Resilience

The first group of coping self-insights, significantly positively related to resilience, were those related to *Time course*, *Growth reappraisal*, and *Strengths*. Although these three coping self-insights were less commonly attained in the actual data, the weighted data that simulated equal likelihood of their observance across the sample revealed statistical significance. These self-insights connote more immediate positive feelings about the self and personal capacity, and may increase a sense of hope, agency, or self-efficacy.

*Strengths* (“understanding the nuanced interactions between individual strengths and the effectiveness of coping strategies or resources”) [13] (p. 11) showed the greatest effect size; however, was rarely attained, with only two participants in the successful condition demonstrating one instance each. Nonetheless, these participants acknowledged positive personal characteristics (i.e., creativity, wisdom, and leadership style) that enabled effective coping.

*Growth reappraisal* (“understanding that stressors, while uncomfortable, also provide an opportunity for growth across the lifespan”) [13] (p. 10) was also positively associated with perceived resilience at 6 months, occurring for only 12 (15%) participants. Participants with these self-insights identified that they, and others, were able to learn from difficult circumstances. For example, “Difficult things and learning to deal with them builds capacity for encountering and dealing with future challenges” (P277, S), “I/the other person can cope with and learn from that [i.e., looking less prepared or even failing]” (P038, U), and “I can turn a negative stressful situation to encourage and teach my team” (P251, S). Hence, these insights demonstrate identification of personal capacity for coping.

A third coping self-insight positively associated with resilience was *Time course* (i.e., “understanding the time course of one’s reactions”) [13] (p. 7). Again, this was one of the less commonly attained insights (*n* = 12, 15%) however those higher in this self-insight recognized an initial reaction to a stressor followed by a period whereby their cognitive, affective, physical, and/or behavioral responses altered in intensity or nature over time. For example, “When a stressful situation happens thoughts and feelings are there quickly. I can see what’s happening and analyse it quite quickly. However! I am much better not to actually respond from the feelings. And wait. Don’t respond immediately. Don’t speak immediately except to give a positive response”. (P142, S) or “When I deal with a challenging situation, I find that in addition to the anxiety or frustration that I feel in situ, I often have an emotional spike two or three days later. Knowing this enables me to prepare for it, and process it better”. (P023, S). Thus, recognizing typical response timelines appears to reveal a personal regulatory capability.

### 3.4.2. Coping Self-Insights Negatively Related to Resilience

Self-insights that were significantly negatively related to perceived resilience included *Interpersonal effect*, *Capacities applied*, *Trigger interpretation* and *Distinct outcomes*. The coping self-insight with the largest negative effect size on resilience 6 months post-training was *Distinct outcomes* (i.e., “understanding the potential for coping strategies to be associated with distinct or even oppositional shorter- and longer-term outcomes”) [13] (p. 11). This coping self-insight was also significantly more likely to be attained by those in the unsuccessful coping self-reflection training condition (see Table S4). Participants commonly identified the use of avoidance strategies that distracted them from stressors immediately, but that did not contribute to their mitigation (e.g., “I did not deal with my emotional or physical stress, just suppressed it for a time”. (P196, U)). Participants also identified problem-focused coping strategies that enabled them to address the stressful situation but which they recognized were unsustainable in the long term (e.g., “Skipping sleep, got through things but not feeling strongly effective by the end […]—short term gain only” (P057, U)). Some participants identified that while strategies they used increased stress in the short term, the benefits paid off later (e.g., “Planning and coordinating created more stress in the short term while trying to problem-solve but relieved stress when completed” (P317, S)). Overall, workbook entries detailed the recognition of short-term strategies that had some immediate benefit in reducing stress, but were either ineffective (e.g., procrastination) or unsustainable in the long term (e.g., increased work hours).

The second coping self-insight negatively related to resilience was *Trigger interpretation* (“interpretations of why these situations induce stress”) [13] (p. 9). Ministry workers referred to their personal values as a core element of many of the interpretations they offered. Some interpretations illustrated the high value placed on interpersonal relationships (e.g., “I deeply care that people understand my intentions and my desire to care for them. The thought that I had been neglectful of a team member was deeply upsetting for me and I couldn’t rest until the relationship issues were resolved”; (P317, S)), others’ perceptions (e.g., “I need to acknowledge [my] dependency on people’s positive perception”. (P102, U)), and their responses to criticism (e.g., “I take personal criticism very deeply—especially when unjust/unfair—I can’t seem to let it go”. (P138, U)). Other interpretations of triggers revealed a recognition of a discrepancy between personal values, particularly regarding achievement and competence in the context of pastoral ministry (e.g., “I struggle with these situations because I want to be seen to be competent and to achieve…” (P236, S)). Thus, recognition of a discrepancy between values and personal interpretations of behavior was associated with lower down-stream resilience.

*Capacities applied* (i.e., “acknowledging the coping strategies, resources, or beliefs typically applied in the coping process”) was also negatively related to perceived resilience. Identification in workbook entries revealed that participants recognized how they tend to respond to stressors, 67% of which suggested some incongruity between those tendencies and their preferred or ideal responses (e.g., “When I get stressed, I focus on myself and my feelings and I often sideline the people I love” (P275, U)). Hence, self-insights revealing incongruity were associated with lower resilience.

*Interpersonal effect* (i.e., “understanding the influence of one’s personal reactions on the behavior of others and vice versa”) [13] (p. 7) was negatively related to resilience. Examples demonstrated how participants’ responses affected someone else (e.g., “Considered how I’m impacting others […] I need to moderate my feelings to match the mood/circumstances” (P078, U)) and how others’ behaviors or emotions affected them (e.g., “the way people speak impacts the way I typically respond” (P068, U)). Comments suggest that while this is a self-insight derived from the self-awareness reflective process, there appears to be some deficit-focused self-evaluation associated with this insight. That is, there was a recognized discordance between their actual response and how they would prefer to have acted.

Further to these four self-insights, results for *Capacity modification* approached statistical significance, suggesting a negative association with perceived resilience. This newly introduced self-insight revealed participants’ understanding of “aspects of resilient capacities that can be modified to support resilience in the future”. That is, participants attaining this self-insight identified gaps in their coping repertoire requiring modification to aid future resilient outcomes. For example, “Learn some skills to ensure that I don’t project in my mind a possible outcome—when this may not be the reality” (P011, U). Although non-significant, it is noteworthy that this self-insight that highlights a discrepancy in beliefs, coping strategies, or resources is also suggestive of a negative association with perceived resilience.

### 3.4.3. Coping Self-Insights Not Significantly Related to Resilience

A third group of seven coping self-insights (i.e., *Stressor reactions*, *Personal values*, *Trigger patterns*, *Capacity effectiveness*, *Desired responses*, *Anticipated effect*, and *Resource congruence*) was not significantly associated with perceived resilience. As aforementioned, *Capacity effectiveness* identifies self-insights into particular resilient capacities that are either helpful or unhelpful for the individual. For example, both of these self-insights fit the category: “I’ve realised that when I step-back and take a break, things don’t feel as intense and tend to fall into place…” (P317, S) and “Being pragmatic with emotional pain is unhelpful” (P153, S). Exploration of the other six coping self-insights within participants’ qualitative data suggests a similar pattern where the definition of the self-insight captures a self-understanding that may equally relate to personal capacity and agency, to a gap or discrepancy in resilient capacities, or to a neutral point of acceptance. For example, within Stressor reactions, the relationship between the self-insight and the participant’s perceived resilience is likely to depend on the nature of the reaction relationship. For example, a helpful reaction (e.g., “I have […] realised my anxious anticipation […] is heading to certain expectations and behaviours which I have been able to catch and recast in a hopefully more healthy way”. (P186, S)) is likely to lead to a higher sense of self-efficacy in contrast to an unhelpful one (e.g., “My feelings affected my ability to be empathetic, patient and loving” (P192, U)). Similarly, for *Trigger patterns* the self-insight might be positively associated with perceived resilience if it produces a sense of agency (e.g., “Expecting and understanding this seasonal funk makes it much less severe” (P023, S)). However, recognition of a trigger pattern might be negatively related to perceived resilience if it highlights a discrepancy in values or desired behaviors (e.g., “That my flawed nature rears its head for propensity to be angry when my boys disobey/are going crazy” (P160, S)). Also encountered within the qualitative data were examples of a participant attaining a self-insight that suggested a neutral acceptance of the situation (e.g., “I was reminded of how immediate and deep my somatic/emotional response is to a request for help”. (186, S)). The findings revealed that coping self-insights, such as those related to stressor reactions or trigger patterns, do not neatly fall into positive or negative categories. Instead, they represent a broader and more nuanced understanding of one’s own capacities that may have contributed to a neutral or non-significant association with perceived resilience.

## 4. Discussion

### 4.1. Summary of Findings

This study aimed to understand the association of specific coping self-insights with perceived resilience and used a mixed-methods approach necessitated by the quantitizing of qualitative coping self-insights. Participants’ workbook entries demonstrated evidence of the coping insights proposed by the framework [12,13] and provided refinements to the coping self-insights. Two insights required a broadening of their definition and a new coping self-insight was introduced—*Capacity modification*. Although it was hypothesized that attainment of any coping self-insight would be associated with perceived resilience, the findings provided only partial support for this prediction.

Two sets of coping self-insights were found to have opposite relationships with perceived resilience. *Growth reappraisal*, *Strengths*, and *Time course* were positively associated with perceived resilience 6 months post-reflection. Each of these coping self-insights, identified through the coding process, conveyed recognition of personal capacity in some respect: through acknowledgement of opportunity for growth and personal characteristics that support achievement, or self-understanding that negative states will pass. Mirroring recent research identifying most critical insight dimensions for resilience [26], this set of coping self-insights appears to relate to capability, perhaps engendering a sense of optimism, agency, and self-efficacy.

Another set of self-insights related to the identification of coping limitations (*Interpersonal effect*, *Capacities Applied*, *Trigger Interpretation*, and *Distinct outcomes*) were associated with lower perceived resilience 6 months post-reflection, with *Capacity modification* approaching significance. This association was strongest when a coping deficit was recognized explicitly (i.e., *Distinct outcomes* self-insights that acknowledge distinct or oppositional effects of strategies used). However, discrepancies were also implicit (i.e., *Trigger interpretation*, *Interpersonal effect*, and *Capacities applied*) where no direct judgement of coping was made, but the admission of vulnerability to a type of stressor, or recognition of undesirable behaviors, may imply failure to cope well.

A third set of coping self-insights was not found to be significantly related to perceived resilience 6 months post-reflection (i.e., *Stressor reactions*, *Personal values*, *Trigger patterns*, *Capacity effectiveness*, *Desired responses*, *Anticipated effect*, and *Resource congruence*).

Thus, these findings suggest that coping self-insights highlighting coping capability supported perceived resilience; however, attainment of discrepancy-related coping self-insights diminished perceptions of resilience.

### 4.2. Theoretical and Applied Implications

On face value, our findings suggest that self-reflection strategies that engender self-insights into coping capacities predicted higher perceived resilience, while others that engender self-insights into coping limitations predicted lower perceived resilience. Indeed, drawing from the Systematic Self-Reflection model [6], this was the basis for our hypothesis that attainment of *any* self-insight would be associated with perceived resilience. However, we propose that these findings may inform refinement of the Systematic Self-Reflection model by revealing two possible pathways for resilience development via self-reflection across time. First, attainment of self-insights that highlight existing coping capabilities and opportunities for growth fosters resilient beliefs (e.g., self-efficacy and optimism) and mid-term positive perceptions of resilience. Second, attainment of self-insights relating to discrepancies or deficits in coping act as feedback that can result in functional modifications to coping behaviors but take time to reform. Inherent in this second pathway is the experience of discomfort as people develop self-insight into their current coping limitations. This discomfort stemming from an acknowledgement of personal limitation is expected to manifest as a potentially temporary diminishment in perceived resilience. That is, “I realize I need to find better ways of coping, perhaps I’m not as resilient as I thought”. Nevertheless, as recognized by the Systematic Self-Reflection model [6], it is this discomfort that is expected to drive functional coping adaptations. It is also conceivable that those who have higher psychological distress (i.e., lower resilient capacities) are more likely to pay more attention to their coping deficits.

The finding that both helpful and unhelpful instances can emerge within the same type of self-insight may mean that the person’s perception of their own resilience may increase or decline for the same self-insight category creating a neutral or non-significant association. For instance, recognizing a trigger pattern may provide a sense of agency, which positively influences perceived resilience. However, that same recognition may also underscore a struggle or vulnerability lowering one’s perception of resilience.

In terms of behavioral change, the former pathway reflects a capability belief strategy, and the later pathway reflects a self-monitoring and feedback strategy [27]. Several questions emerge from these possible dual pathways: (1) Does highlighting capabilities via self-reflection promote only a positive appraisal of one’s resilience, but no ‘real’ contribution to capability? (2) Does highlighting potential gaps in capabilities contribute to actual deteriorations in resilience or just provide a more critical/realistic appraisal of one’s resilience? (3) Does highlighting coping limitations promote the refinement and expansion of capacities in the longer term? (4) Do actual coping deficits and capacity limitations contribute to personal understanding of these discrepancies and deficits? and (5) Is it possible to simultaneously attain a sense of effectiveness and recognize our limitations in coping, or do these two approaches clash in their functionality?

In relation to the first question, if reflecting on evidence of capabilities enhances resilient beliefs, such as a sense of coping self-efficacy, then this may engender an actual increase in resilient outcomes. A recent synthesis of 277 interventions found that, in 23 studies, focusing on past success as a technique was strongly linked to beliefs about capabilities as the mechanism that brings about actual change [27]. Coping self-efficacy is frequently regarded as a key capacity for resilience insofar as those who believe in their capabilities to achieve desired goals are more likely to persevere in stressful situations [28]. Other research supports this notion. In a study of patients with breast cancer, Karademas et al. (2023) found that coping self-efficacy mediated the effects of trait resilience on quality of life and psychological symptoms, and high levels of each were associated with better outcomes [29]. Further, in a study by Dolcos et al. (2021), coping self-efficacy mediated the relationship between religious coping and both anxiety and depression [30]. In this way, we propose that attainment of coping capability self-insights may promote actual resilient outcomes via the enhancement of coping self-efficacy.

Regarding the second, third, and fourth questions, this research identified four self-insights negatively related to resilience that highlighted coping limitations. These insights encompassed explicit self-evaluations of ineffective coping strategy selection, and implicit disappointment in capacity application, interpersonal effects of reactions, and in interpretations of why these situations trigger stress. Given the evidence for the first question, a focus solely on coping failure may lessen the likelihood of actual rather than just perceived resilient outcomes, at least in the mid-term, through a reduction in self-efficacy, optimism, or hope [28].

Numerous lines of research underscore the significance of feedback as a pivotal change catalyst. In a collaborative study by Michie and colleagues (2021) [27], involving 105 global behavior change experts, it was unanimously agreed that feedback was a conduit through which disparities between conduct and valued goals function as a behavior modification technique. In our context, feedback materializes as prompt and contextualized self-assessments of coping actions, potentially propelling behavioral change. As suggested in Michie and colleagues’ previous (2011) [31] work, there may be limitations to the effects of this feedback where individuals lack a sense of efficacy for change (e.g., perceiving inadequate capability for change), lack a clear pathway to change (e.g., lacking strategies for improved coping), experience overwhelming circumstances (e.g., facing an excessive need for change), or encounter motivational deficits (e.g., lacking desire for change). Nevertheless, feedback concerning incongruous coping approaches may be necessary for the assimilation and integration of functional modifications to coping practices, even if the transformation process requires an extended duration or entails multiple unsuccessful endeavors. Consequently, we propose that the acquisition of self-insights highlighting disparities might temporarily reduce perceived resilience.

It may also be possible that mental health status effects the process of strengthening resilience through attainment of self-insights by self-reflection. Indeed, in proposing the Systematic Self-Reflection model, Crane et al. (2019) [6] have indicated that the process might be circular such that those who are depressed (i.e., lower in resilient capacities) may be more likely to gain self-insights related to their coping discrepancies and deficits because they are more salient. This may also be reinforced by seeking help from mental health professionals where there is a deficit focus. Rumination, as a transdiagnostic pathological process, may moderate the attainment of both capability- and deficit-related self-insights [3,32]. However, people who already perceive themselves to be lower in resilience may be more aware of their coping limitations which, in turn, may emphasize that self-perception.

Given these considerations, we recommend implementing and evaluating interventions that use of a combination of successful and unsuccessful coping reflections. These may: (i) offset a deterioration in perceived resilience, and (ii) increase self-insights that enhance the refinement and development of coping strategies through an iterative, albeit potentially protracted, process [13].

This leads to the last question regarding whether two pathways may be combined in an intervention to enhance outcomes in the longer term. We posit that the integration of structured reflection on both successful and unsuccessful coping experiences may yield greater benefits than just one focus of self-reflection. There may also be potential benefit in the temporally staggered introduction of different self-reflection strategies. For example, enhancing the salience of existing coping capabilities may support resilient beliefs such as self-efficacy initially, followed by a more critical self-evaluation of coping limitations to promote behavioral modification. This might be tested by conducting trials with conditions using combinations of successful and unsuccessful coping self-reflection. Clinically, it could also be tested using behavioral experiments in CBT-based interventions for stressed patient groups.

### 4.3. Limitations

The primary constraint of this study pertains to the potentially restricted applicability of the findings due to the specialized nature of the sampled group’s occupational context. Given that individuals in ministry roles typically dedicate time to introspectively ponder personal values and their practical embodiment, this subset might be inherently inclined to acquire self-insights linked with values to a greater extent than a more generalized population sample. Notwithstanding this, a notable coherence in the ranking pattern of coping self-insights emerged between this sample and a cohort of Officer Cadets [12]. It is also noteworthy that this study was conducted in 2020 when the global pandemic had far-reaching impacts on mental health outcomes [33]. As such, the context in which this study was conducted may also influence the generalizability of the findings. Further investigations are necessary to ascertain whether these outcomes can be extended to demographically balanced community samples and individuals in different professions and contexts.

Another limitation pertains to the distribution of participants exhibiting the coping self-insights. Considering the limited number of participants who attained the three self-insights linked to heightened perceived resilience, we employed inverse proportionality weighting as a corrective measure. Additionally, we included the pre-training resilience variable as a predictor in the hierarchical regression analyses delineated in this study. However, it remains necessary to exercise caution when interpreting these findings, as they warrant future investigation.

Further limitations arise from speculative nature of the conclusions drawn from this study’s findings. That is, in suggesting possible dual pathways, we propose a significant role for self-efficacy which is untested. Addition of a measure of self-efficacy (as well as other resilient belief variables such as optimism and hope) in future studies would aid understanding of these possible mechanisms. Moreover, follow-up measures beyond six months were not assessed in this study. Longitudinal studies extending to two or more years would enable understanding of how long it takes to embed behavioral change resulting from discrepancy-related self-insights.

## 5. Conclusions

The outcomes from this study suggest that self-reflection may engender distinct self-insights that lead to downstream resilient outcomes. In one pathway, self-reflection highlighting coping capability appears to foster mid-term perceptions of resilience. In another pathway, self-reflection provides coping discrepancy feedback that can result in modification to coping behaviors, but which is expected to take time to embed and to enhance perceived resilience. We recommend further exploration into context-specific coping self-insights, as well as the use of and on-going refinement of self-reflection resilience training.

## Figures and Tables

**Table 1:** Coping Self-Insights, Definitions, Examples, Instances, and (Rank Order) of Instances.

Coping Self-Insight	Definition	Example	Instances (Rank)
**Self-awareness**			
Time course	Understanding the time course of one’s reactions	“Giving myself the time and space to move beyond my reactive emotions usually means that I see things with greater clarity” (P201, S)	12 (13)
Stressor reactions	Understanding the inter-relationships between one’s reactions	“My feelings can be really strong. I usually […] know exactly what I’m feeling and can blame others quickly if they’re feelings I don’t want” (P038, U)	17 (11)
Interpersonal effect	Understanding the influence of one’s personal reactions on the behavior of others and vice versa	“When my wife gets upset at being away from family, I can get frustrated with her and begin to distance myself”. (P153, S)	29 (9)
Personal values	Understanding whether one’s response to a stressor moves them towards or away from their personal values	“I don’t think I showed humility and self-control which are values I aspire to have” (P061, U)	75 (2)
Capacities applied	Acknowledging the coping strategies, resources, or beliefs typically applied in the coping process	“I can tend to remove myself from relationship when stressed […] and become narrowly focused on task over people” (P205, S)	40 (6)
**Trigger identification**			
Trigger patterns	Understanding the overarching patterns of triggers across time and contexts	“I find special occasions raise quite a lot of sadness and regret for me” (P190, S)	35 (8)
Trigger interpretation	Interpretations of why these situations induce stress	“I struggle with these situations because I want to be seen to be competent and to achieve…” (P236, S)	42 (5)
**Growth Reappraisal**	Understanding that stressors, while uncomfortable, also provide an opportunity for growth across the lifespan	“Difficult things and learning to deal with them builds capacity for encountering and dealing with future challenges” (P277, S)	12 (13)
**Evaluation**			
Capacity effectiveness	Understanding the effectiveness of particular coping strategies, resources or beliefs for personal coping	“Praying to God helped me remember what’s important” (P089, S)	77 (1)
Distinct outcomes	Understanding the potential for coping strategies to be associated with distinct or even oppositional shorter- and longer-term outcomes	“Skipping sleep, got through things but not feeling strongly effective by the end […]—short term gain only” (P057, U)	17 (11)
Strengths	Understanding the nuanced interactions between individual strengths and the effectiveness of coping strategies or resources	“I know that I enjoy producing creative work, so I was confident that I would be able to complete the task” (P023, S)	2 (15)
Desired responses	Understanding of desired responses or outcomes that align with personal values	“I want to rely and trust in God—deal with feelings of grief and loss rather than respond to feelings of being burdened” (P239, U)	52 (4)
**Future-focus**			
Anticipated effect	Understanding the anticipated effect of resilient capacities applied in the future.	“Get outside for some exercise to get a better headspace” (P276, U)	73 (3)
Capacity modification (New)	Understanding aspects of resilient capacities (e.g., skills, resources) that can be modified to support resilience in the future	“I’ve learned that I need a way to reduce my stress levels in the moment of stress, not just afterward” (P159, U)	22 (10)
Resource congruence	Understanding the congruence between the type and source of coping resources available, and the anticipated needs of the individual in their future stressor context	“Other ministry friends—there’s a sense in which I can ask them questions about what it is they struggle with” (P071, U)	38 (7)

## Data Availability

The data that support the findings of this study are available from the corresponding author, K.J.B., upon reasonable request.

## Supplementary Materials

The following supporting information can be downloaded at: https://www.mdpi.com/article/10.3390/bs14111018/s1. Table S1. Summary of use of measures. Table S2. Means and standard deviations of study variables by condition. Table S3. Correlational matrix (Pearson’s Point Biserial and Cramer’s V) of study variables resilience, general self-insight and coping self-inisght instances. Shaded correlations (above the Diagonal) relate to the unsuccessful coping condition, and unshaded correlations (under the Diagonal) relate to the successful coping condition. Table S4. Binary logistic regressions of condition on coping self-insights as outcome variables. Table S5. Unweighted and inverse probability weighted sample sizes for hierarchical regressions on perceived resilience with coping self-insight instances. Table S6. Hierarchical regression analyses of weighted coping self-insight instances ever on perceived resilience.

## References

[1] Hill C.E., Castonguay L.G., Angus L., Arnkoff D.B., Barber J.P., Bohart A.C., Borkovec T.D., Bowman E.A., Caspar F., Gibbons M.B., Castonguay L.G., Hill C. (2007). Insight in Psychotherapy: Definitions, Processes, Consequences, and Research Directions. Insight in Psychotherapy.

[2] Grant A.M., Franklin J., Langford P. (2022). The self-reflection and insight scale: A new measure of private self-consciousness. Soc. Behav. Personal..

[3] Bucknell K.J., Kangas M., Crane M.F. (2022). Adaptive self-reflection and resilience: The moderating effects of rumination on insight as a mediator. Personal. Individ. Differ..

[4] Cowden R.G., Meyer-Weitz A. (2016). Self-reflection and self-insight predict resilience and stress in competitive tennis. Soc. Behav. Personal..

[5] Kalisch R., Baker D.G., Basten U., Boks M.P., Bonanno G.A., Brummelman E., Chmitorz A., Fernàndez G., Fiebach C.J., Galatzer-Levy I. (2017). The resilience framework as a strategy to combat stress-related disorders. Nat. Hum. Behav..

[6] Crane M.F., Searle B.J., Kangas M., Nwiran Y. (2019). How resilience is strengthened by exposure to stressors: The systematic self-reflection model of resilience strengthening. Anxiety Stress Coping.

[7] Di Stefano G., Gino F., Pisano G., Staats B.R. (2023). Learning by Thinking: How Reflection Can Spur Progress Along the Learning Curve.

[8] Ellis S., Davidi I. (2005). After-event reviews: Drawing lessons from successful and failed experience. Appl. Psychol..

[9] Takano K., Tanno Y. (2009). Self-rumination, self-reflection, and depression: Self-rumination counteracts the adaptive effect of self-reflection. Behav. Res. Ther..

[10] Trapnell P.D., Campbell J.D. (1999). Private self-consciousness and the five-factor model of personality: Distinguishing rumination from reflection. Personal. Soc. Psychol..

[11] Lysaker P.H., Kukla M., Vohs J.L., Schnakenberg Martin A.M., Buck K.D., Hasson Ohayon I. (2019). Metacognition and recovery in schizophrenia: From research to the development of metacognitive reflection and insight therapy. Exp. Psychopathol..

[12] Falon S.L., Hoare S., Kangas M., Crane M.F. (2022). The coping insights evident through self-reflection on stressful military training events: Qualitative evidence from self-reflection journals. Stress Health.

[13] Falon S.L., Kangas M., Crane M.F. (2021). The coping insights involved in strengthening resilience: The Self-Reflection and Coping Insight Framework. Anxiety Stress Coping.

[14] Falon S.L., Karin E., Boga D., Gucciardi D.F., Griffin B., Crane M.F. (2021). A clustered-randomized controlled trial of a self-reflection resilience-strengthening intervention and novel mediators. Occup. Health Psychol..

[15] Bucknell K.J., Kangas M., Karin E., Crane M.F. (2024). A randomized controlled trial comparing the effects of self-reflective writing focused on successful and unsuccessful coping experiences on resilience. Stress Health.

[16] Ryba T.R., Wiltshire G., North J., Ronkainen N.J. (2022). Developing mixed methods research in sport and exercise psychology: Potential contributions of a critical realist perspective. Int. J. Sport Exerc. Psychol..

[17] Smith B.W., Dalen J., Wiggins K., Tooley E., Christopher P., Bernard J. (2008). The brief resilience scale: Assessing the ability to bounce back. Int. J. Behav. Med..

[18] Roberts C., Stark P. (2008). Readiness for self-directed change in professional behaviours: Factorial validation of the Self-Reflection and Insight Scale. Med. Educ..

[19] Sandelowski M., Voils C.I., Knafl G. (2009). On Quantitizing. Mix. Methods Res..

[20] Proudfoot K. (2023). Inductive/Deductive Hybrid Thematic Analysis in Mixed Methods Research. Mix. Methods Res..

[21] McLean K.C., Thorne A. (2003). Late adolescents’ self-defining memories about relationships. Dev. Psychol..

[22] Landis J.R., Koch G.G. (1997). The measurement of observer agreement for categorical data. Biometrics.

[23] Braun V., Clarke V., Cooper H., Camic P.M., Long D.L., Panter A.T., Rindskopf D., Sher K.J. (2012). Thematic analysis. APA Handbook of Research Methods in Psychology.

[24] Rosenbaum P.R., Rubin D.B. (1983). The central role of the propensity score in observational studies for causal effects. Biometrika.

[25] Shiba K., Kawahara T. (2021). Using propensity scores for causal inference: Pitfalls and tips. Epidemiology.

[26] Crane M.F., Hoare S., Kangas M., Gucciardi D.F., Karin E. (2024). A coping self-insight scale for adults: Development and preliminary psychometric properties. Anxiety Stress Coping.

[27] Michie S., Johnston M., Rothman A.J., de Bruin M., Kelly M.P., Carey R.N., Bohlen L.E., Groarke H.N., Anderson N.C., Zink S. (2021). Developing an evidence based online method of linking behaviour change techniques and theoretical mechanisms of action: A multiple methods study. Health Serv. Deliv. Res..

[28] Hamill S.K. (2003). Resilience and self-efficacy: The importance of efficacy beliefs and coping mechanisms in resilient adolescents. Colgate Univ. J. Sci..

[29] Karademas E.C., Simos P., Pat-Horenczyk R., Roziner I., Mazzocco K., Sousa B., Stamatakos G., Tsakou G., Cardoso F., Frasquilho D. (2023). The interplay between trait resilience and coping self-efficacy in patients with breast cancer: An international study. Clin. Psychol. Med. Settings.

[30] Dolcos F., Hohl K., Hu Y., Dolcos S. (2021). Religiosity and resilience: Cognitive reappraisal and coping self-efficacy mediate the link between religious coping and well-being. Relig. Health.

[31] Michie S., van Stralen M.M., West R. (2011). The behaviour change wheel: A new method for characterising and designing behaviour change interventions. Implement. Sci..

[32] Nolen-Hoeksema S., Watkins E.R. (2011). A heuristic for developing transdiagnostic models of psychopathology: Explaining multifinality and divergent trajectories. Perspect. Psychol. Sci..

[33] Veer I.M., Riepenhausen A., Zerban M., Wackerhagen C., Puhlmann L.M., Engen H., Köber G., Bögemann S.A., Weermeijer J., Uściłko A. (2021). Psycho-social factors associated with mental resilience in the Corona lockdown. Transl. Psychiatry.

