# Supplementary material for: A Mixed-Methods Study Exploring Coping Self-Insights Associated with Resilience
Source: Behav Sci (Basel). 2024 Nov 1;14(11):1018. doi: 10.3390/bs14111018 (PMC11590910; doi:10.3390/bs14111018)
Supplement: Supplementary file 1 [file behavsci-14-01018-s001.zip › behavsci-3182582-supplementary.pdf]

Supplementary Materials

Materials and Methods

Table S1

Summary of Use of Measures

Scale	Used in this paper	Used in parent study [15]
Brief Resilience Scale		
Pre-training	✓	✓
Post-training		✓
3 months post-training		✓
6 months post-training	✓	✓
Self-Reflection & Insight Scale		
Insight factor, pre-training	✓	✓
Coping Self-Insight Instances	✓	

S2.2 Example Workbook Questions and Responses

Participants in this study wrote responses to ten self-reflective questions once a week for five weeks. Two example questions and several example responses are included below.

Example Question A: What strategies did you use to reduce your levels of stress or cope with this event?

Example Response #1: "Clarified with another person what happened; Took out some time to reflect, process and pray about the matter; Debriefed with my wife and a colleague; Asked to meet with [person] to discuss/ understand/ learn (this hasn't happened yet)." (P037, S)

Example Response #2: "Prayer; Mindfulness to deal with my thoughts and emotions; Processed my feelings somewhat with my boss; Reflected on why I feel as I do – blocking the achievement value for me is the issue I have with the person involved" (P058, U)

Example Response #3: "I had practiced some breathing techniques at night during previous evenings and prayed. On the day itself the encounter was a chance encounter so there was little opportunity to reduce stress or cope – I just dived into it. I had previously spoken with my wife about it." (P136, S)

Example Response #4: ""Escape"-ism - fled the environment… but without resolution. I tried to "push-on" from home but inability to focus just made worse. To "feel" like I had done something I sent messages of 'complaint' to people who should/ could have addressed outstanding issues... but this would not change or fix anything. Binged on social media and news." (P193, U)

Example Question B: What might you learn about yourself or what useful knowledge, skills, or capabilities did you develop from this experience?

Example Response #1: “That praying before the call is not enough, I need to also pray during the call and breathing in and out plus speaking to myself – remind myself in the moment.” (P019, S)

Example Response #2: “To make a habit of having the hard conversations with people and that people, some at least, appreciate it.” (P029, S)

Example Response #3: “I’m not good at predicting the future accurately, but it is my default when stressed to expect catastrophe and not be able to see the situation with clarity.” (P040, U)

Example Response #4: “I learnt that procrastinating does not solve problems, but sometimes you need to talk a problem out to stop the procrastination.” (P097, S)

Example Response #5: “That I don't handle conflict well - not enough guts to confront - but also afraid of negative outcome knowing the other person is going through a lot of stress themselves.” (P102, U)

Example Response #6: “To continue to be more reflective and not get too busy – so move onto the next thing” (P140, U)

Example Response #7: “I need to turn to prayer sooner.” (P196, U)

Example Response #8: “That I need to feel sympathised with, particularly by my wife.” (P209, S)

Results

Table S2

Means and Standard Deviations of Study Variables by Condition

	Unsuccessful		Successful	
	Mean	*SD*	Mean	*SD*
Resilience (Pre-training)	3.45	0.79	3.38	0.64
Resilience (6-months post-training)	3.51	0.79	3.62	0.55
Self-Insight (Pre-training)	4.54	0.68	4.75	0.45
Coping Self-Insight Instances				
Time course	0.08	0.27	0.23	0.43
Stressor reactions	0.30	0.46	0.13	0.34
Interpersonal effect	0.30	0.46	0.44	0.50
Personal values	0.95	0.22	0.95	0.22
Capacities applied	0.53	0.51	0.49	0.51
Trigger patterns	0.43	0.50	0.46	0.51
Trigger interpretation	0.53	0.51	0.54	0.51
Growth reappraisal	0.08	0.27	0.23	0.43
Capacity effectiveness	0.95	0.22	1.00	0.00
Distinct outcomes	0.32	0.47	0.10	0.31
Strengths	0.00	0.00	0.05	0.22
Desired responses	0.72	0.45	0.59	0.50
Anticipated effect	0.90	0.30	0.95	0.22
Capacity modification	0.38	0.49	0.18	0.39
Resource congruence	0.48	0.51	0.49	0.51

Table S3

Correlational Matrix (Pearson's Point Biserial and Cramer's V) of Study Variables Resilience, General Self-Insight and Coping Self-Insight Instances. Shaded Correlations (above the Diagonal) Relate to the Unsuccessful Coping Condition, and Unshaded Correlations (under the Diagonal) Relate to the Successful Coping Condition.

	1	2	3	4	5	6	7	8	9	10	11	12	13	14	15	16	17	18
1		.687**	.419**	-.041	-.025	.195	.180	.018	-.017	.178	.262	-.260	-.010	-	.087	.030	-.288	.163
2	.596**		.367*	-.004	.193	.037	.143	.011	.035	-.054	.381*	-.070	-.135	-	.077	.160	-.305	.301
3	.410**	.129		-.079	.045	-.001	.013	.096	.065	-.148	.216	-.206	.036	-	-.091	-.105	-.332*	-.022
4	-.253	-.088	.052		*.021*	*.228*	*.065*	*.109*	*.053*	*.109*	*.279*	*.065*	*.208*	-	*.037*	*.095*	*.025*	*.109*
5	-.132	-.181	-.007	*.336**		*.048*	*.150*	*.186*	*.320**	*.295*	*.435***	*.150*	*.128*	-	*.037*	*.218*	*.282*	*.251*
6	.024	-.152	-.039	*.113*	*.028*		*.150*	*.186*	*.011*	*.295*	*.021*	*.150*	*.105*	-	*.086*	*.218*	*.056*	*.076*
7	-.318*	-.125	-.019	*.127*	*.089*	*.030*		*.011*	*.197*	*.241*	*.065*	*.053*	*.086*	-	*.372**	*.688***	*.178*	*.218*
8	.083	-.284	.035	*.290*	*.240*	*.178*	*.239*		*.210*	*.098*	*.081*	*.011*	*.088*	-	*.199*	*.017*	*.194*	*.098*
9	-.359*	-.421**	-.109	*.103*	*.414**	*.327**	*.018*	*.538***		*.008*	*.331**	*.197*	*.159*	-	*.190*	*.118*	*.170*	*.195*
10	-.182	-.315	.027	*.141*	*.047*	*.503***	*.018*	*.079*	*.444***		*.109*	*.011*	*.232*	-	*.311**	*.017*	*.323**	*.203*
11	-.173	-.162	-.006	*.011*	*.154*	*.009*	*.127*	*.047*	*.225*	*.263*		*.065*	*.208*	-	*.037*	*.095*	*.025*	*.299*
12	-	-	-	-	-	-	-	-	-	-	-		*.086*	-	*.141*	*.306*	*.178*	*.011*
13	-.361*	-.413**	-.082	*.185*	*130*	*.214*	*.079*	*.178*	*.365**	*.313*	*.216*	-		-	*.069*	*.302*	*.124*	*.088*
14	-.417**	-.017	-.205	*.149*	*.259*	*.030*	*.054*	*.227*	*.251*	*.215*	*.424***	-	*.305*		-	-	-	-
15	-.138	-.183	.102	*.209*	*.164*	*.418***	*.279*	*.021*	*.354**	*.273*	*.209*	-	*.110*	*.194*		*.168*	*.014*	*.137*
16	-.226	-.125	-.315	*.127*	*.089*	*.204*	*.473***	*.006*	*.215*	*.251*	*.127*	-	*.079*	*.054*	*.279*		*.258*	*.150*
17	-.179	-.098	-.188	*.220*	*.020*	*.142*	*.109*	*.189*	*.165*	*.031*	*.098*	-	*.158*	*.109*	*.118*	*.109*		*.090*
18	-.052	-.259	.133	*.075*	*.393**	*.029*	*.227*	*.282*	*.332**	*.182*	*.075*	-	*.178*	*.239*	*.187*	*.227*	*.055*	

Note. 1 = Brief Resilience Scale (pre-training); 2 = Brief Resilience Scale, (6 months post-training); 3 = Insight factor of Self-Reflection and Insight Survey (pre-training); 4 = Time course; 5 = Stressor reactions; 6 = Interpersonal effect; 7 = Personal values; 8 = Capacities applied; 9 = Trigger patterns; 10 = Trigger interpretation; 11 = Growth reappraisal; 12 = Capacity effectiveness; 13 = Distinct outcomes; 14 = Strengths; 15 = Desired responses; 16 = Anticipated effect; 17 = Capacity modification; 18 = Resource congruence.

Italics represents Cramer's V for correlation of two categorial variables.

$^{**}p < .01$; $^{*}p < .05$

Table S4

Binary Logistic Regressions of Condition on Coping Self-Insights as Outcome Variables

Coping Self-Insight	B	*SE*	Wald x^2	*p*-value	Exp (B)	LCI	UCI
Time course	1.308	0.71	3.391	0.066	3.7	0.919	14.893
Stressor reactions	-1.07	0.59	3.283	0.07	0.343	0.108	1.091
Interpersonal effect	0.589	0.473	1.556	0.212	1.803	0.714	4.553
Personal values	-0.027	1.026	0.001	0.979	0.974	0.13	7.278
Capacities applied	-0.151	0.45	0.113	0.737	0.86	0.356	2.078
Trigger patterns	0.148	0.453	0.107	0.744	1.16	0.477	2.82
Trigger interpretation	0.054	0.451	0.014	0.905	1.056	0.436	2.555
Growth reappraisal	1.308	0.71	3.391	0.066	3.7	0.919	14.893
Capacity effectiveness	18.258	6436.0	0	0.998	8.50e7	0	.
Distinct outcomes	-1.438	0.627	5.269	**0.022**	0.237	0.07	0.81
Strengths	18.285	6355.1	0	0.998	8.73e7	0	.
Desired responses	-0.606	0.481	1.59	0.207	0.545	0.212	1.4
Anticipated effect	0.721	0.897	0.645	0.422	2.056	0.354	11.928
Capacity modification	-1.009	0.53	3.626	0.057	0.365	0.129	1.03
Resource congruence	0.049	0.45	0.012	0.914	1.05	0.434	2.539

Note. **Bold** indicates *p*-value <.05

Table S5

Unweighted and Inverse Probability Weighted Sample Sizes for Hierarchical Regressions on Perceived Resilience with Coping Self-Insight Instances

Coping Self-Insight	Cases	Unweighted sample size	Weighted sample size
Time course	12	79	247
Stressor reactions	17	79	249
Interpersonal effect	29	79	224
Personal values	75	79	154
Capacities applied	40	79	239
Trigger patterns	35	79	219
Trigger interpretation	42	79	205
Growth reappraisal	12	79	247
Capacity effectiveness	77	79	156
Distinct outcomes	17	79	249
Strengths	2	79	259
Desired responses	52	79	183
Anticipated effect	73	79	152
Capacity modification	22	79	233
Resource congruence	38	79	231

Table S6

Hierarchical Regression Analyses of Weighted Coping Self-Insight Instances Ever on Perceived Resilience

Coping Self-Insight Effect	Step 1 β	Step 1 *SE*	Step 1 *p*	Step 1 *t*	Step 2 β	Step 2 *SE*	Step 2 *p*	Step 2 *t*
Time course								
(Constant)		.03	<.001	130.60		.04	<.001	86.44
Condition	.24	.06	<.001	5.27	.22	.06	<.001	4.61
Age	-.12	<.01	.009	-2.63	-.14	<.01	.004	-2.92
Gender	-.29	.06	<.001	-5.81	-.29	.06	<.001	-5.91
Resilience T1	.85	.04	<.001	15.94	.87	.04	<.001	16.26
Self-insight T1	-.14	.07	.016	-2.42	-.14	.07	.016	-2.44
Time course					.10	.06	**.017**	2.40
Stressor reactions								
(Constant)		.04	<.001	102.32		.05	<.001	68.04
Condition	-.08	.07	.095	-1.67	-.07	.07	.129	-1.52
Age	-.09	<.01	.072	-1.80	-.09	<.01	.077	-1.78
Gender	-.23	.08	<.001	-4.68	-.23	.08	<.001	-4.55
Resilience T1	.62	.05	<.001	12.30	.62	.05	<.001	12.28
Self-insight T1	.19	.07	<.001	3.72	.19	.07	<.001	3.66
Stressor reactions					.02	.08	.663	0.44
Interpersonal effect								
(Constant)		.03	<.001	114.23		.04	<.001	84.93
Condition	.14	.07	.014	2.48	.15	.07	.007	2.74
Age	-.08	<.01	.171	-1.37	-.08	<.01	.151	-1.44
Gender	-.23	.07	<.001	-4.11	-.22	.07	<.001	-4.08
Resilience T1	.62	.05	<.001	10.72	.63	.05	<.001	10.98
Self-insight T1	.15	.07	.011	2.56	.14	.07	.015	2.45
Interpersonal effect					-.10	.07	**.034**	-2.14

Coping Self-Insight	Step 1				Step 2			
Effect	β	*SE*	*p*	*t*	β	*SE*	*p*	*t*
Personal values								
(Constant)		.04	<.001	86.63		.04	<.001	85.39
Condition	.13	.09	.042	2.05	.13	.09	.041	2.07
Age	-.04	<.01	.501	-0.68	-.04	<.01	.564	-0.58
Gender	-.16	.09	.012	-2.55	-.17	.09	.011	-2.59
Resilience T1	.65	.07	<.001	9.40	.65	.07	<.001	9.41
Self-insight T1	.05	.08	.449	0.76	.05	.08	.470	0.72
Personal values					.05	.26	.455	0.75
Capacities applied								
(Constant)		.03	<.001	109.67		.04	<.001	84.42
Condition	.03	.07	.527	0.63	.02	.07	.613	0.51
Age	-.06	<.01	.236	-1.19	-.06	<.01	.216	-1.24
Gender	-.09	.07	.067	-1.84	-.08	.07	.119	-1.56
Resilience T1	.69	.05	<.001	13.57	.69	.05	<.001	13.68
Self-insight T1	.04	.07	.372	0.90	.05	.07	.325	0.99
Capacities applied					-.10	.09	**.038**	-2.09
Trigger patterns								
(Constant)		.04	<.001	97.63		.05	<.001	74.62
Condition	.05	.08	.391	0.86	.05	.08	.408	0.83
Age	-.15	<.01	.014	-2.48	-.15	<.01	.012	-2.52
Gender	-.15	.08	.009	-2.63	-.15	.08	.013	-2.52
Resilience T1	.53	.06	<.001	8.34	.52	.06	<.001	8.11
Self-insight T1	.09	.08	.146	1.46	.09	.08	.141	1.48
Trigger patterns					-.03	.09	.589	-0.54

Coping Self-Insight Effect	Step 1 β	*SE*	*p*	*t*	Step 2 β	*SE*	*p*	*t*
Trigger interpretation								
(Constant)		.03	<.001	110.85		.04	<.001	92.07
Condition	.18	.07	.002	3.22	.18	.07	.001	3.28
Age	-.04	<.01	.461	-0.74	-.05	<.01	.411	-0.82
Gender	-.16	.07	.005	-2.83	-.14	.07	.012	-2.54
Resilience T1	.70	.05	<.001	12.27	.71	.05	<.001	12.50
Self-insight T1	-.02	.06	.724	-0.35	-.03	.06	.568	-0.57
Trigger interpretation					-.13	.08	**.010**	-2.60
Growth reappraisal								
(Constant)		.04	<.001	98.44		.05	<.001	66.75
Condition	-.12	.09	.033	-.214	-.19	.09	.002	-3.21
Age	-.07	<.01	.214	-1.25	-.04	<.01	.468	-0.73
Gender	.05	.08	.363	0.91	.07	.08	.223	1.22
Resilience T1	.58	.06	<.001	9.08	.58	.06	<.001	9.35
Self-insight T1	.01	.09	.900	0.13	-.04	.09	.559	-0.59
Growth reappraisal					.18	.08	**<.001**	3.58
Capacity effectiveness								
(Constant)		.04	<.001	88.06		.04	<.001	87.55
Condition	.12	.08	.068	1.84	.11	.09	.091	1.70
Age	-.05	<.01	.430	-0.79	-.05	<.01	.470	-0.73
Gender	-.16	.09	.012	-2.55	-.17	.09	.008	-2.68
Resilience T1	.64	.07	<.001	9.49	.66	.07	<.001	9.58
Self-insight T1	.06	.08	.422	0.80	.06	.08	.376	0.89
Capacity effectiveness					.07	.37	.229	1.21

Coping Self-Insight	Step 1				Step 2			
Effect	β	*SE*	*p*	*t*	β	*SE*	*p*	*t*
Distinct outcomes								
(Constant)		.03	<.001	100.03		.05	<.001	76.28
Condition	.09	.07	.065	1.85	.01	.08	.813	0.24
Age	-.01	<.01	.809	-0.24	-.05	<.01	.315	-1.01
Gender	.03	.07	.611	0.51	.07	.07	.163	1.40
Resilience T1	.74	.05	<.001	12.87	.68	.05	<.001	11.49
Self-insight T1	.03	.06	.629	0.48	.03	.06	.542	0.61
Distinct outcomes					-.17	.08	**<.001**	-3.62
Strengths								
(Constant)		.03	<.001	108.26		.03	<.001	114.44
Condition	.39	.07	<.001	6.41	.13	.07	.018	2.38
Age	-.12	<.01	.101	-1.65	-.15	<.01	.012	-2.53
Gender	-.42	.06	<.001	-5.76	-.49	.05	<.001	-8.24
Resilience T1	.47	.05	<.001	6.22	1.03	.05	<.001	12.72
Self-insight T1	-.00	.07	.959	-0.05	.03	.06	.608	0.51
Strengths					.88	.08	**<.001**	10.89
Desired responses								
(Constant)		.04	<.001	100.00		.04	<.001	88.00
Condition	.12	.08	.046	2.01	.12	.08	.051	1.96
Age	-.02	<.01	.774	-0.29	-.02	<.01	.752	-0.32
Gender	-.13	.08	.030	-2.18	-.13	.08	.033	-2.15
Resilience T1	.68	.06	<.001	10.82	.68	.06	<.001	10.76
Self-insight T1	.01	.07	.829	0.22	.01	.07	.824	0.22
Desired responses					-.01	.10	.799	-0.26

Coping Self-Insight Effect	Step 1 β	Step 1 *SE*	Step 1 *p*	Step 1 *t*	Step 2 β	Step 2 *SE*	Step 2 *p*	Step 2 *t*
Anticipated effect								
(Constant)		.04	<.001	86.45		.04	<.001	85.03
Condition	.12	.09	.071	1.82	.11	.09	.090	1.70
Age	-.05	<.01	.455	-0.75	-.05	<.01	.470	-0.73
Gender	-.16	.09	.014	-2.50	-.16	.09	.012	-2.54
Resilience T1	.65	.07	<.001	9.49	.65	.07	<.001	9.52
Self-insight T1	.06	.08	.408	0.83	.07	.08	.326	0.99
Anticipated effect					.09	.21	.162	1.40
Capacity modification								
(Constant)		.04	<.001	100.22		.05	<.001	74.13
Condition	.23	.07	<.001	4.43	.21	.07	<.001	4.06
Age	-.10	<.01	.070	-1.82	-.09	<.01	.113	-1.59
Gender	-.21	.08	<.001	-3.66	-.21	.08	<.001	-3.80
Resilience T1	.64	.05	<.001	11.51	.62	.05	<.001	11.16
Self-insight T1	-.06	.06	.327	-0.98	-.08	.06	.190	-1.31
Capacity modification					-.10	.08	.057	-1.91
Resource congruence								
(Constant)		.03	<.001	108.49		.04	<.001	81.62
Condition	-.03	.07	.553	-0.59	-.03	.07	.568	-0.57
Age	-.05	<.01	.316	-1.01	-.05	<.01	.298	-1.04
Gender	-.10	.07	.070	-1.82	-.11	.07	.050	-1.97
Resilience T1	.63	.05	<.001	11.41	.63	.05	<.001	11.35
Self-insight T1	.10	.06	.082	1.75	.10	.06	.077	1.78
Resource congruence					.05	.09	.329	0.98
